# Association Between Baseline Depressive Symptoms and Clinical Characteristics, Biomarker Profiles, and Clinical Outcomes in Patients With Peripheral Arterial Disease Undergoing Revascularization: A Prospective Cohort Study

**DOI:** 10.7759/cureus.106829

**Published:** 2026-04-11

**Authors:** Alexandros Giosdekos, Christos Bakoyiannis, Emmanouil Rizos, Ioannis D Kakisis

**Affiliations:** 1 Department of Vascular Surgery, Attikon University General Hospital, Athens, GRC; 2 First Department of Surgery, National and Kapodistrian University of Athens, Laikon General Hospital, Athens, GRC; 3 Department of Psychiatry, Attikon University General Hospital, Athens, GRC

**Keywords:** depression, inflammation, major adverse limb events, peripheral arterial disease, revascularization

## Abstract

Background

Depressive symptoms are a common comorbidity in patients with peripheral arterial disease (PAD) and have been associated with adverse cardiovascular outcomes. However, their relationship with inflammatory and neuroendocrine biomarkers and their prognostic significance following lower extremity revascularization remains incompletely defined. This study aimed to evaluate whether baseline clinically significant depressive symptoms are associated with clinical characteristics, biomarker profiles, and 24-month clinical outcomes in patients with PAD undergoing revascularization.

Methods

This prospective, dual-center cohort study included 150 consecutive patients with symptomatic PAD undergoing lower limb revascularization (January 2022-December 2023). Depressive symptoms were assessed using the Patient Health Questionnaire-9 (PHQ-9), with a score ≥10 indicating clinically significant depressive symptoms. Baseline levels of C-reactive protein (CRP), interleukin-6 (IL-6), and cortisol were measured. The primary endpoint was major adverse limb events (MALE) at 24 months. Kaplan-Meier and Cox regression analyses were performed, with adjustment for clinically relevant covariates, including disease severity.

Results

Of 150 patients, 80 (53.3%) had clinically significant depressive symptoms. Smoking (65.0% vs 37.1%; p = 0.001) and prior cardiovascular events (46.3% vs 25.7%; p = 0.01) were more frequent in this group, which also had a higher prevalence of advanced PAD (Rutherford 4-6). CRP, IL-6, and cortisol levels were higher in patients with depressive symptoms (all p < 0.001). MALE occurred in 37.5% vs 11.4% (p < 0.001). Event-free survival was lower in patients with depressive symptoms (log-rank p < 0.001). In multivariable analysis, depressive symptoms remained associated with MALE (HR: 4.03, 95% CI: 1.84-8.83; p < 0.001). However, given the baseline imbalance in disease severity and the observational design, these findings should be interpreted with caution.

Conclusions

Clinically significant depressive symptoms are associated with a distinct clinical and biological profile and with worse clinical outcomes in patients undergoing lower extremity revascularization for PAD. These findings suggest a potential prognostic relationship; however, the observed associations may be influenced by disease severity and other confounding factors, and further studies are required to clarify underlying mechanisms and causality.

## Introduction

Peripheral arterial disease (PAD) is a major manifestation of systemic atherosclerosis, affecting over 200 million people worldwide, with prevalence rising sharply with age and a substantial proportion of asymptomatic cases [1,2]. Clinically, PAD ranges from intermittent claudication to chronic limb-threatening ischemia (CLTI), which is associated with high risks of limb loss and cardiovascular mortality [1,3,4].

Depression, affecting approximately 5% of adults globally, is a frequent comorbidity in cardiovascular disease and is associated with increased mortality and poorer functional outcomes [2,5-9]. In PAD, depressive symptoms have been linked to reduced functional capacity and worse clinical outcomes [6,8,10-12].

Potential mechanisms underlying this association include systemic inflammation and neuroendocrine dysregulation, with elevated C-reactive protein (CRP), interleukin-6 (IL-6), and cortisol implicated in both depression and atherosclerosis [7,11,13]. Depression in PAD has been associated with increased risk of major adverse limb events (MALE), amputation, and mortality [6,11,12], although the underlying biological pathways remain incompletely understood.

Therefore, the aim of the present study was to evaluate whether baseline clinically significant depressive symptoms, as assessed by the Patient Health Questionnaire-9 (PHQ-9), are associated with (i) baseline clinical characteristics, (ii) inflammatory and neuroendocrine biomarker profiles, and (iii) clinical outcomes, including MALE, following lower extremity revascularization in patients with PAD.

The primary objective was to assess the association between clinically significant depressive symptoms and MALE at 24 months, while secondary objectives included associations with baseline clinical characteristics and biomarker levels. Given the potential impact of disease severity on both biomarkers and outcomes, analyses were designed to account for clinically relevant confounders, including Rutherford classification. The study was designed to assess prognostic associations rather than causal relationships.

We hypothesized that patients with clinically significant depressive symptoms would have (i) a higher burden of adverse clinical characteristics, (ii) higher levels of inflammatory and neuroendocrine biomarkers, and (iii) an increased risk of MALE and reduced event-free survival during follow-up.

## Materials and methods

This prospective, dual-center observational cohort study was conducted at two tertiary vascular centers. Consecutive adult patients (≥18 years) with symptomatic PAD undergoing lower limb revascularization (endovascular or open) were enrolled between January 2022 and December 2023. Inclusion criteria were symptomatic PAD requiring revascularization and the ability to provide informed consent and complete baseline assessment. Exclusion criteria included systemic acute or chronic inflammatory disease, active malignancy, severe psychiatric disorders other than depression (e.g., psychotic or bipolar disorders), incomplete data, technically unsuccessful revascularization, or inability to complete follow-up.

Disease severity was classified according to the Rutherford classification, which stratifies PAD into clinical stages ranging from asymptomatic disease (category 0) to intermittent claudication (categories 1-3) and chronic limb-threatening ischemia (categories 4-6), based on clinical presentation, including claudication, rest pain, and tissue loss [14].

Depressive symptoms were assessed at baseline using the PHQ-9, a validated self-administered screening instrument. The PHQ-9 consists of nine items corresponding to the diagnostic criteria for major depressive disorder, each scored from 0 to 3, yielding a total score ranging from 0 to 27. A threshold of ≥10 was used to define clinically significant depressive symptoms [15,16].

Venous blood samples were obtained via standard venipuncture prior to revascularization under standardized preanalytical conditions. All samples were collected in the morning (between 07:00 and 10:00), after an overnight fast, with patients in a seated position following a rest period of at least 15 minutes. Patients receiving systemic corticosteroids were excluded. Serum levels of CRP, IL-6, and cortisol were measured using standard immunoassay-based laboratory methods at the participating institutions.

Due to skewed distributions, biomarker values were log-transformed for statistical analyses. Group comparisons and corresponding p-values were derived from analyses performed on log-transformed data, while descriptive data are presented as mean ± standard deviation.

The primary endpoint was the first occurrence of MALE within 24 months, defined as a composite of target limb reintervention, major amputation, or death attributable to PAD. PAD-related death was adjudicated based on review of clinical records, imaging data, and follow-up documentation by the treating clinical teams. Secondary endpoints included the individual components of the composite and all-cause mortality. Time-to-event was calculated from the date of revascularization to the first event or censoring. Patients were followed for up to 24 months through scheduled clinical visits and review of medical records.

Continuous variables are presented as mean ± standard deviation. Comparisons between groups were performed using the Student's t-test or Mann-Whitney U test, as appropriate based on data distribution. Categorical variables were compared using the chi-square test or Fisher's exact test when appropriate. Event-free survival was evaluated using Kaplan-Meier analysis and compared using the log-rank test.

Univariable and multivariable Cox proportional hazard regression analyses were performed to assess associations with outcomes. Multivariable models were prespecified and included clinically relevant covariates (age, sex, current smoking, prior cardiovascular events, and Rutherford classification). Given the number of outcome events, the number of variables included in the model was limited to reduce the risk of overfitting. Proportional hazards assumptions were verified using Schoenfeld residuals.

A two-sided p-value <0.05 was considered statistically significant. All analyses were performed using Statistical Product and Service Solutions (SPSS, version 29.0; IBM SPSS Statistics for Windows, Armonk, NY).

**Figure 1:**
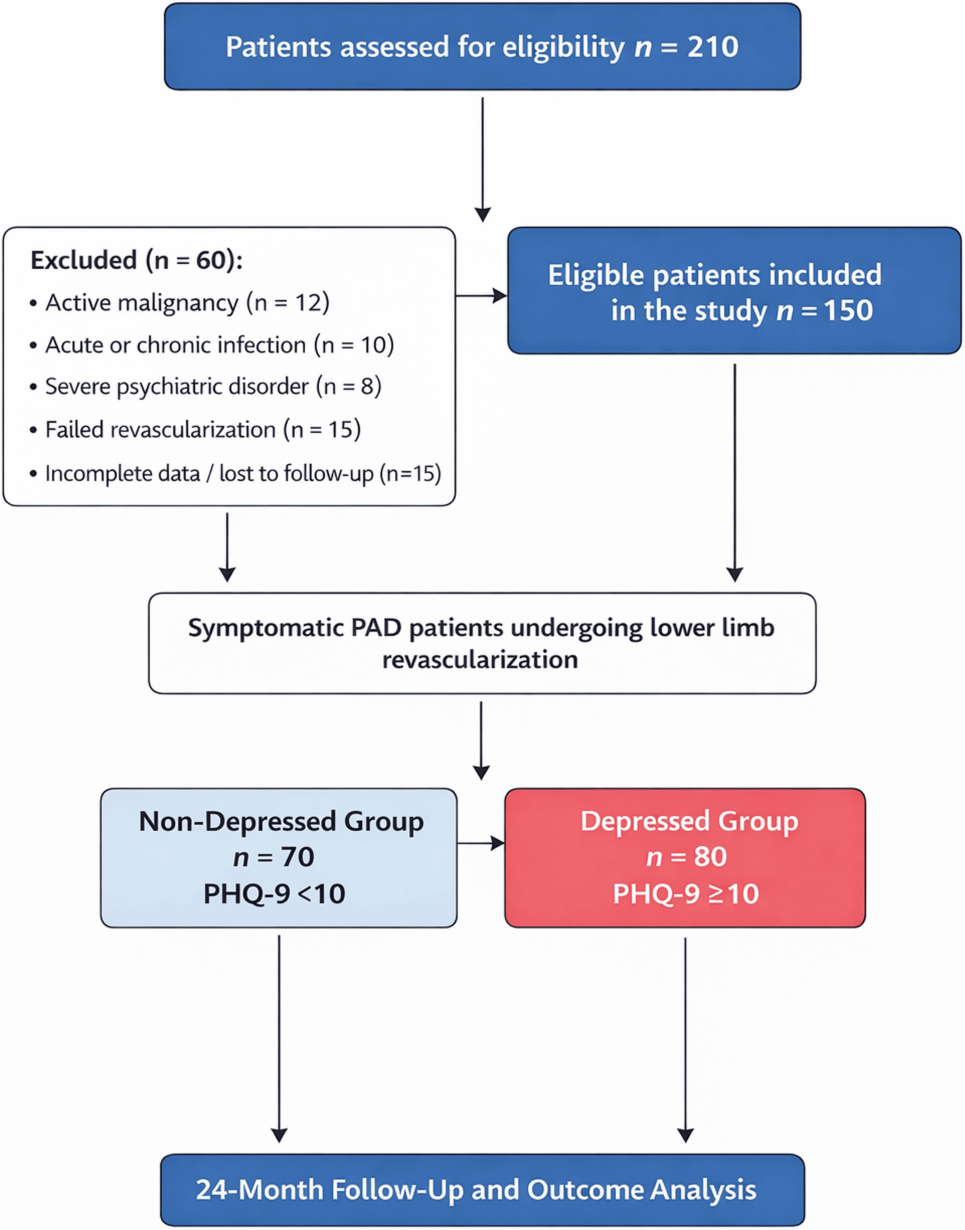
Study flowchart of patient enrollment and follow-up Flow diagram illustrating patient selection, inclusion and exclusion criteria, and final cohort allocation into depressed and non-depressed groups, with 24-month follow-up and outcome assessment.

The study was designed to evaluate prognostic associations rather than causal relationships. The study was approved by the institutional review boards of both participating centers, and all patients provided written informed consent in accordance with the Declaration of Helsinki. The study flow is presented in Figure 1.

## Results

A total of 150 patients were included, of whom 80 (53.3%) were classified as having clinically significant depressive symptoms and 70 (46.7%) as non-depressed based on baseline PHQ-9 assessment (cut-off ≥10). Baseline characteristics are presented in Table 1. Age and sex distribution were similar between groups. Current smoking was more frequent in patients with depressive symptoms (65.0% vs 37.1%; p = 0.001), as was a history of prior cardiovascular events (46.3% vs 25.7%; p = 0.01). Patients with depressive symptoms had a higher prevalence of advanced disease according to Rutherford classification (Rutherford 4-6: 58.7% vs 11.4%; p < 0.001), whereas non-depressed patients were more frequently classified as Rutherford 0-3 (Table 1).

**Table 1:** Baseline demographic and clinical characteristics of the study population according to depression status Data are presented as mean ± standard deviation or number (percentage). The statistical test used for each comparison is indicated in the table. PHQ-9: Patient Health Questionnaire-9; PAD: peripheral arterial disease

Variable	Total (n=150)	Non-depressed (n=70)	Depressed (n=80)	Test	p-value
Age (years)	68.0 ± 10.6	66.6 ± 9.7	69.2 ± 11.1	Student's t-test	0.136
Male sex, n (%)	89 (59.3%)	43 (61.4%)	46 (57.5%)	Chi-square test	1.00
Current smoking, n (%)	78 (52.0%)	26 (37.1%)	52 (65.0%)	Chi-square test	0.001
Prior cardiovascular events, n (%)	55 (36.7%)	18 (25.7%)	37 (46.3%)	Chi-square test	0.01
PHQ-9 score	12.3 ± 9.1	4.0 ± 2.1	19.8 ± 5.7	Mann-Whitney U	<0.001
Rutherford 0-3	95 (63.3%)	62 (88.6%)	33 (41.3%)	Chi-square test	<0.001
Rutherford 4-6	55 (36.7%)	8 (11.4%)	47 (58.7%)	Chi-square test	<0.001

PHQ-9 scores were higher in the depressed group compared to the non-depressed group (19.8 ± 5.7 vs 4.0 ± 2.1; p < 0.001). The proportion of patients classified as Rutherford 0-3 was higher in the non-depressed group (88.6% vs 41.3%), whereas Rutherford 4-6 was more frequent in the depressed group (58.7% vs 11.4%; p < 0.001).

Baseline Biomarker Profile

**Figure 2:**
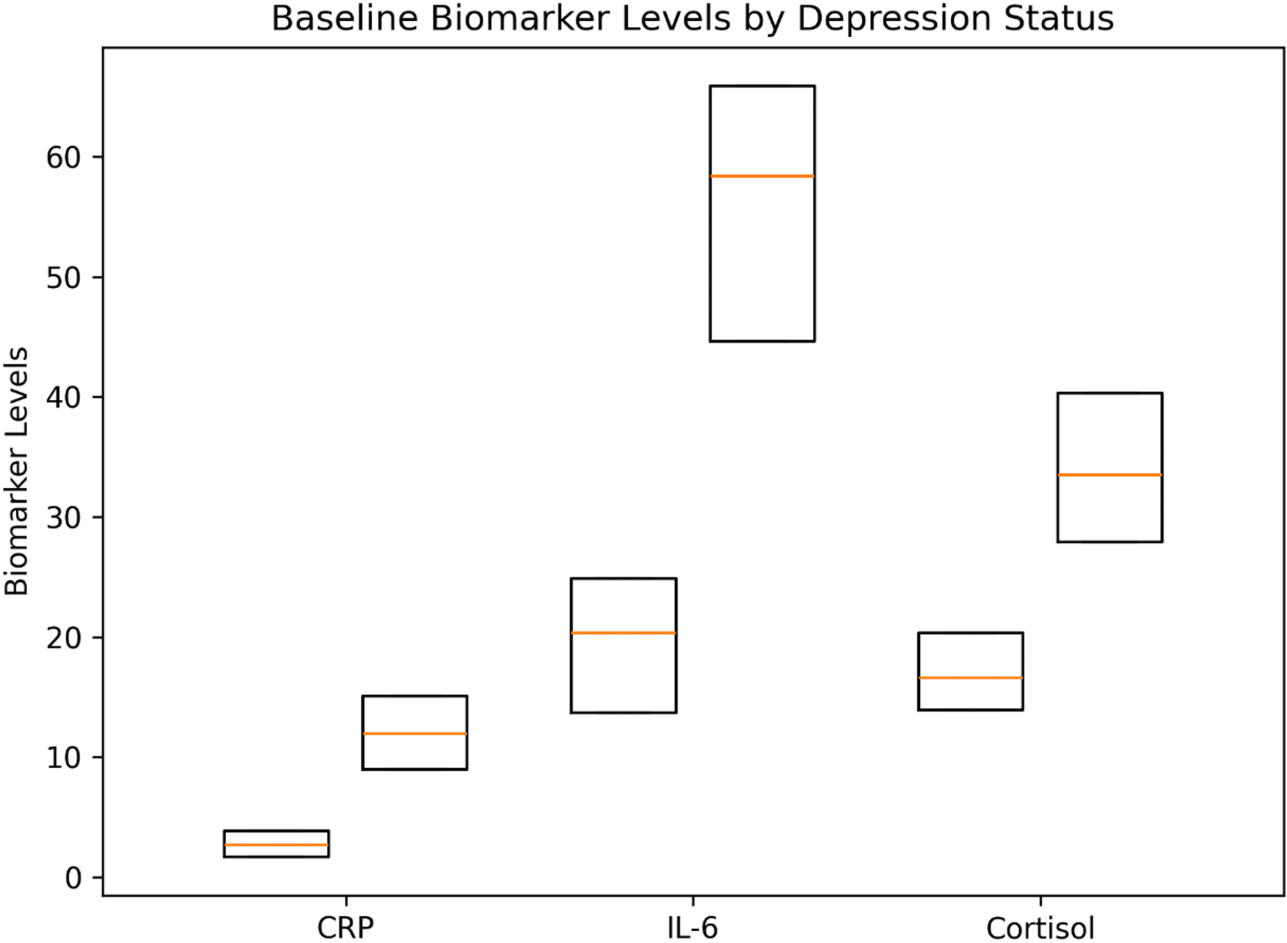
Baseline inflammatory and neuroendocrine biomarker levels according to depression status Baseline serum levels of CRP, IL-6, and cortisol stratified by depression status. Biomarker levels were higher in the depressed group (all p < 0.001). Values are presented as median (interquartile range). CRP: C-reactive protein; IL-6: interleukin-6

Baseline levels of inflammatory and neuroendocrine biomarkers are presented in Table 2 and Figure 2. CRP, IL-6, and cortisol levels were higher in patients with clinically significant depressive symptoms compared to the non-depressed group (all p < 0.001). Given the differences in disease severity between groups, these findings should be interpreted descriptively.

**Table 2:** Baseline inflammatory and neuroendocrine biomarker levels according to depression status Data are presented as median (interquartile range). P-values were derived from comparisons performed on log-transformed values. CRP: C-reactive protein; IL-6: interleukin-6

Biomarker	Non-depressed (n=70)	Depressed (n=80)	Test	p-value
CRP (mg/L)	2.64 (1.68-3.84)	11.91 (8.95-15.10)	Student's t-test on log-transformed values	<0.001
IL-6 (pg/mL)	20.35 (13.66-24.89)	58.41 (44.61-65.91)	Student's t-test on log-transformed values	<0.001
Cortisol (μg/dL)	16.58 (13.90-20.30)	33.50 (27.88-40.34)	Student's t-test on log-transformed values	<0.001

Clinical Outcomes

During the 24-month follow-up, the primary endpoint (MALE) occurred in 30 of 80 patients (37.5%) in the depressed group and in eight of 70 patients (11.4%) in the non-depressed group (χ² test; p < 0.001) (Table 3).

**Table 3:** Clinical outcomes at 24 months according to depression status Data are presented as number (percentage). The statistical test used for each comparison is indicated in the table. The primary endpoint was defined as a composite of reintervention, major amputation, or PAD-related death. PAD-related death was adjudicated based on clinical records and follow-up data. MALE: major adverse limb events; PAD: peripheral arterial disease

Outcome	Non-depressed (n=70)	Depressed (n=80)	Test	p-value
MALE (primary endpoint)	8 (11.4%)	30 (37.5%)	Chi-square test	<0.001
Reintervention	5 (7.1%)	18 (22.5%)	Chi-square test	0.009
Major amputation	3 (4.3%)	10 (12.5%)	Fisher's exact	0.088
PAD-related death	2 (2.9%)	7 (8.8%)	Fisher's exact	0.175
All-cause mortality	4 (5.7%)	11 (13.8%)	Fisher's exact	0.171

Reintervention occurred in 18 of 80 patients (22.5%) in the depressed group and in five of 70 patients (7.1%) in the non-depressed group (χ² test; p = 0.009). Major amputation occurred in 10 patients (12.5%) in the depressed group and in three patients (4.3%) in the non-depressed group (Fisher's exact test; p = 0.088). PAD-related death occurred in seven patients (8.8%) and two patients (2.9%), respectively (Fisher's exact test; p = 0.175), while all-cause mortality occurred in 11 patients (13.8%) and 4 patients (5.7%), respectively (Fisher's exact test; p = 0.171; Table 3).

**Figure 3:**
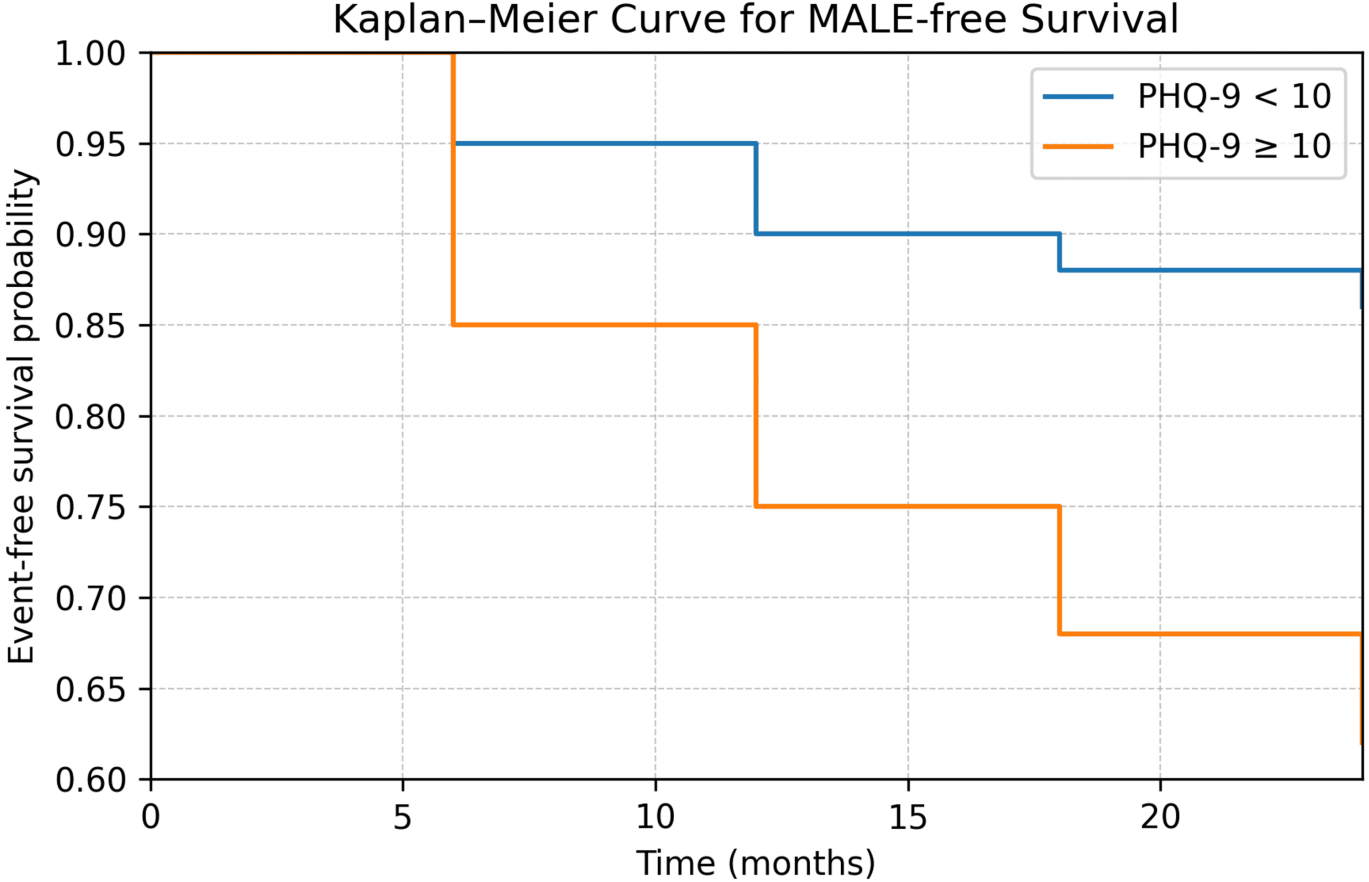
Kaplan-Meier curves for event-free survival free from major adverse limb events (MALE) according to depressive symptom status Patients were stratified based on the presence of clinically significant depressive symptoms (PHQ-9 ≥10). Event-free survival over 24 months differed between groups, with lower survival probabilities observed in patients with clinically significant depressive symptoms (log-rank χ²(1) = 19.41; p < 0.001).

Event-free survival was lower in patients with clinically significant depressive symptoms compared to non-depressed patients (log-rank χ²(1) = 19.41; p < 0.001; Figure 3).

Cox Regression Analysis

In univariable Cox regression analysis, clinically significant depressive symptoms were associated with an increased risk of major adverse limb events (HR: 3.85, 95% CI: 1.77-8.41; p < 0.001).

In multivariable Cox regression analysis, adjusted for age, sex, current smoking, prior cardiovascular events, and Rutherford classification, clinically significant depressive symptoms remained associated with MALE (HR: 4.03, 95% CI: 1.84-8.83; p < 0.001). Age was not associated with the outcome (HR: 0.98, 95% CI: 0.96-1.01; p = 0.27).

**Figure 4:**
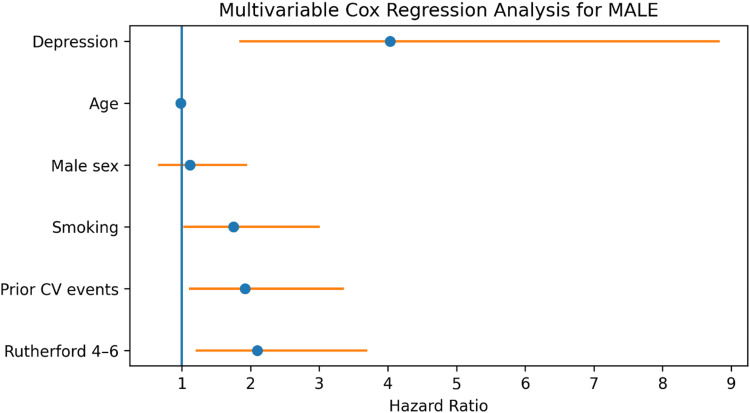
Multivariable Cox regression analysis for major adverse limb events (MALE) Forest plot presenting hazard ratios (HR) with 95% confidence intervals for variables included in the multivariable Cox regression model. Depression remained independently associated with increased risk of MALE after adjustment for age, sex, smoking status, prior cardiovascular events, and disease severity (Rutherford classification).

Results are presented in Table 4 and Figure 4.

**Table 4:** Multivariable Cox regression analysis for major adverse limb events (MALE) Multivariable analysis was adjusted for age, sex, current smoking status, prior cardiovascular events, and disease severity (Rutherford classification). HR: hazard ratio; CI: confidence interval

Variable	HR	95% CI	p-value
Depression (yes vs no)	4.03	1.84-8.83	<0.001
Age (per year)	0.98	0.96-1.01	0.27
Male sex	1.12	0.65-1.95	0.68
Current smoking	1.75	1.02-3.01	0.04
Prior cardiovascular events	1.92	1.10-3.36	0.02
Rutherford 4-6 vs 0-3	2.10	1.20-3.70	0.01

## Discussion

Principal Findings

In this prospective, dual-center cohort of patients undergoing lower extremity revascularization for PAD, clinically significant depressive symptoms, as assessed by the PHQ-9, were associated with differences in clinical characteristics, biomarker profiles, and clinical outcomes. Specifically, patients with clinically significant depressive symptoms exhibited a higher prevalence of smoking and prior cardiovascular events, higher levels of inflammatory and neuroendocrine biomarkers, and higher rates of MALE. In multivariable analysis, clinically significant depressive symptoms remained associated with MALE after adjustment for selected clinically relevant covariates. These findings should be interpreted as prognostic associations and do not imply causality.

Depressive Symptoms and Baseline Clinical Profile

The present findings suggest that patients with clinically significant depressive symptoms had a higher burden of traditional cardiovascular risk factors, particularly smoking and prior cardiovascular events. This pattern is consistent with prior evidence linking depressive symptoms with adverse health behaviors and increased cardiovascular risk [6-9,11-13,17,18]. These findings highlight the complex interaction between psychological status and cardiovascular disease and support the need for careful consideration of confounding factors when interpreting outcome associations in this population.

Depressive Symptoms, Biomarkers, and Disease Severity

An important observation of the present study is the elevation of inflammatory (CRP, IL-6) and neuroendocrine (cortisol) biomarkers in patients with clinically significant depressive symptoms. These findings are consistent with prior literature suggesting an association between depressive symptoms, low-grade inflammation, and hypothalamic-pituitary-adrenal (HPA) axis dysregulation [13,19,20].

However, interpretation requires caution. Patients with clinically significant depressive symptoms demonstrated a markedly higher proportion of advanced disease (Rutherford 4-6), corresponding to chronic limb-threatening ischemia, which is intrinsically associated with tissue loss, systemic inflammation, and physiological stress. Therefore, the observed biomarker differences may reflect, at least in part, differences in disease severity rather than a direct biological effect of depressive symptoms. This is consistent with prior studies emphasizing the systemic inflammatory burden and functional impairment associated with advanced PAD [2,6,9,11].

Taken together, these findings suggest that depressive symptoms may coexist with, and potentially reflect, a more advanced and inflammatory disease state, rather than acting as an isolated pathophysiological driver.

Depressive Symptoms and Clinical Outcomes

The present study demonstrates an association between clinically significant depressive symptoms and adverse limb outcomes, with higher rates of MALE and reintervention in affected patients. These findings are in line with previous studies reporting worse outcomes among patients with PAD and coexisting depressive symptoms [6,11,12,21].

Although differences in individual endpoints, such as major amputation and mortality, did not reach statistical significance, likely due to limited statistical power, the consistent direction of effect across endpoints supports a potentially clinically relevant association.

Given the observed imbalance in disease severity between groups, additional stratified Kaplan-Meier analyses according to Rutherford classification were performed. These demonstrated a consistent direction of association between clinically significant depressive symptoms and reduced event-free survival across severity strata. However, due to the limited number of events within each subgroup, further multivariable stratified analyses were not performed, and residual confounding by disease severity cannot be excluded.

In addition, death may act as a competing event for limb-related outcomes. The use of standard survival analysis methods (Kaplan-Meier and Cox regression) may therefore overestimate event incidence. Competing risk analyses were not performed and represent an important limitation of the present study.

Prognostic Value and Interpretation

Clinically significant depressive symptoms remained associated with MALE after adjustment for age, sex, smoking, prior cardiovascular events, and disease severity. However, additional relevant confounders, including metabolic comorbidities such as diabetes and obesity, were not systematically incorporated into the analysis and may have influenced the observed associations [6,11,12,21].

Furthermore, the number of outcome events relative to the number of covariates included in the multivariable Cox model was limited, which may affect the stability and precision of the estimates. Although clinically relevant variables were prespecified, some degree of model overfitting and statistical uncertainty cannot be excluded. Therefore, the reported hazard ratios should be interpreted with caution and require confirmation in larger studies.

Overall, these findings support a potential prognostic role of depressive symptoms in PAD, but do not establish a causal relationship.

Clinical Implications

From a clinical perspective, these findings support the potential relevance of psychological assessment in patients with PAD undergoing revascularization. Screening for clinically significant depressive symptoms using simple tools such as the PHQ-9 may help identify patients at higher risk for adverse outcomes who may benefit from closer follow-up and multidisciplinary management [15,16].

However, whether targeted interventions addressing depressive symptoms or associated biological pathways can improve vascular outcomes remains uncertain and warrants further investigation.

Limitations

Several limitations should be acknowledged. First, although the study was prospective, it was observational in nature, and causality cannot be established.

Second, the sample size and number of outcome events were limited, which may have reduced statistical power for secondary endpoints and contributed to relatively wide confidence intervals, indicating imprecision in effect estimates.

Third, clinically significant depressive symptoms were assessed using a self-reported screening instrument (PHQ-9) rather than a formal psychiatric diagnosis. PHQ-9 includes somatic items that may overlap with symptoms of advanced PAD, particularly in patients with chronic limb-threatening ischemia, potentially leading to misclassification.

Fourth, important clinical confounders, including comorbid conditions such as diabetes and obesity, were not systematically included in the analysis. In addition, relevant psychiatric variables, including prior depression, antidepressant or psychotherapeutic treatment, comorbid anxiety or substance use, and pain severity, were not assessed and may represent additional sources of residual confounding.

Fifth, procedural factors, such as revascularization strategy (open versus endovascular), lesion complexity, technical success, and post-procedural medical therapy, were not analyzed in detail, although they may significantly influence outcomes such as reintervention.

Sixth, detailed reporting of follow-up completeness, including loss to follow-up and censoring patterns, was limited, which may influence time-to-event analyses.

Seventh, the definition and ascertainment of prior cardiovascular events were based on available clinical records and may be subject to misclassification.

Eighth, although multivariable adjustment was performed, the number of events relative to the number of covariates was limited, and advanced approaches such as penalized regression, internal validation, and formal assessment of model calibration and collinearity were not performed. Therefore, the stability of the model estimates may be limited.

Finally, competing risk analyses were not performed, and the adjudication of PAD-related death was based on clinical records, which may introduce some degree of subjectivity.

## Conclusions

Depression is associated with increased inflammatory burden and a higher risk of adverse limb outcomes following revascularization in patients with PAD. These findings support the role of depression as a clinically relevant risk factor and underscore the need for integrated management strategies addressing both psychological and vascular components of the disease.
